# Analysis of zero inflated dichotomous variables from a Bayesian perspective: application to occupational health

**DOI:** 10.1186/s12874-021-01427-2

**Published:** 2021-12-12

**Authors:** David Moriña, Pedro Puig, Albert Navarro

**Affiliations:** 1 Department of Econometrics, Statistics and Applied Econometrics, Riskcenter-IREA, Universitat de Barcelona (UB), Avinguda Diagonal 690, 08034 Barcelona, Spain; 2 Centre de Recerca Matemàtica, Universitat Autònoma de Barcelona (UAB), 08193 Cerdanyola del Vallès, Spain; 3 Departament de Matemàtiques, Universitat Autònoma de Barcelona (UAB), Edifici C, Campus de Bellaterra, 08193 Cerdanyola del Vall̀es, Spain; 4 Universitat Autònoma de Barcelona (UAB), Cerdanyola del Vallès, Spain; 5 Unitat de Bioestadística, Facultat de Medicina, Universitat Autònoma de Barcelona, Cerdanyola del Vallès, Spain

**Keywords:** Presenteeism, Bayesian methods, Zero-inflation, Simulation study, Bernoulli mixture models

## Abstract

**Background:**

Zero-inflated models are generally aimed to addressing the problem that arises from having two different sources that generate the zero values observed in a distribution. In practice, this is due to the fact that the population studied actually consists of two subpopulations: one in which the value zero is by default (structural zero) and the other is circumstantial (sample zero).

**Methods:**

This work proposes a new methodology to fit zero inflated Bernoulli data from a Bayesian approach, able to distinguish between two potential sources of zeros (structural and non-structural).

**Results:**

The proposed methodology performance has been evaluated through a comprehensive simulation study, and it has been compiled as an R package freely available to the community. Its usage is illustrated by means of a real example from the field of occupational health as the phenomenon of sickness presenteeism, in which it is reasonable to think that some individuals will never be at risk of suffering it because they have not been sick in the period of study (structural zeros). Without separating structural and non-structural zeros one would be studying jointly the general health status and the presenteeism itself, and therefore obtaining potentially biased estimates as the phenomenon is being implicitly underestimated by diluting it into the general health status.

**Conclusions:**

The proposed methodology is able to distinguish two different sources of zeros (structural and non-structural) from dichotomous data with or without covariates in a Bayesian framework, and has been made available to any interested researcher in the form of the *bayesZIB* R package (https://cran.r-project.org/package=bayesZIB).

**Supplementary Information:**

The online version contains supplementary material available at 10.1186/s12874-021-01427-2.

## Background

In general, zero-inflated models are aimed to addressing the problem that arises from having two different sources that generate the zero values observed in a distribution. In practice, this is due to the fact that the population studied actually consists of two subpopulations: one in which the value zero is by default (structural zero) and the other is circumstantial (sample zero). An example could be the study of sickness presenteeism (SP), i.e. attending work while sick [1]. If it is not previously restricted, the population is made up, among others, of workers who are zero because they have never been sick (structural zeros) and workers who, having been sick, did not attend their work place (sample zeros). Note that the difference is important: roughly the first zero informs us exclusively about the status of health, the second about the exercise of the right to take a sick leave.

The most commonly used zero-inflated models are those that are related to count- ing variables, where it is assumed that the zero value has a dichotomous source that determines whether or not the subject is at risk of suffering the event of interest and another source, only for the individuals at risk, that corresponds to the num- ber of episodes (counts) that have been experienced by each individual at risk. In this context, the most common available models would be the well known Zero- Inflated Poisson (ZIP) and Negative Binomial (ZINB). A good introduction to the mathematical properties of these models can be found in [2], and they have been used in many fields such as quality control ([3]), epidemiology ([4]) or medicine ([5]) among many others. Some guidelines on how to proceed when dealing with count outcomes potentially overdispersed or zero-inflated have been published recently ([6, 7]), based on classical procedures like Vuong’s test ([8]) to check for overdis- persion ([9]) and zero-inflation ([10]), although these guides cannot be applied to the case studied here due to the dichotomous nature of the outcome. In general, zero-inflated models can be expressed as

1 $${\displaystyle \begin{array}{c}P\left(Y=0\right)=g+\left(1-g\right)\cdot f(0)\\ {}P\left(Y=j\right)=\left(1-g\right)\cdot f(j),j>\mathrm{0}\end{array}}$$

where *g* is the structural zero probability and *f* (0) is the zero probability of an appropriate distribution (Poisson, negative binomial or Bernoulli as in our case).

In practice, zero-inflated models with both dichotomous sources (a mixture of two Bernoulli random variables, one with probability of success *ω* and the other with probability of success *p*) have received far less attention. This is due, in large part, to the fact that the resulting distribution is once again a Bernoulli with probability.

of success *ω* · *p*, so that the proportion of structural zeros (1 − *ω*) and sample zeros (1 − *p*) are indistinguishable from the point of view of frequentist statistics. However, from the Bayesian perspective and using known reasonable information about these proportions, it is possible to distinguish the two sources of zeros and estimate *ω* and *p*.

Some authors have recently suggested, in other areas such as the classification or identification of images, the usage of Bernoulli-mixture models, based on numerical algorithms such as Expectation-Maximization (EM) to estimate the parameters [11, 12], given the complexity of the likelihood functions involved. In these cases, however, the inclusion of covariates or adjustment variables is virtually impossible. Also in other areas there are some recent developments in a similar line, such as [13].

In this article we illustrate the use of Zero Inflated Bernoulli (ZIB) models by means of a real dataset on SP, and the results obtained are compared with those of adjusted logistic regressions on the total population or only in those individuals at risk. In the literature, the SP registry is carried out in a self-reported way, asking about the episodes in the last year and later, recorded in a dichotomized way (no SP: 0 episodes; yes SP: 1 or more episodes). The justification for this dichotomization is fundamentally based on two aspects: one, the possible memory bias; second, the excessive influence of workers who report a very high number of episodes.

## Methods

**Fig. 1:**
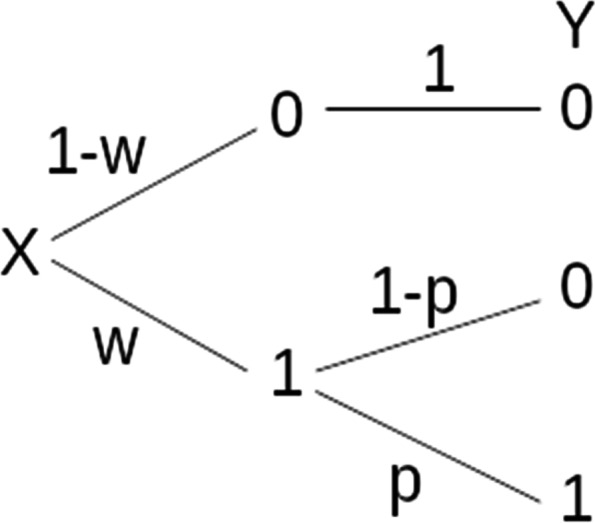
Model schema. Only variable Y is observed

Let *Y* be the variable that indicates occurrence of the phenomenon under study. The proposed model has a probability function defined by

2 $$P\;\left(Y=0\right)=\left(1-\omega\right)+\omega\cdot\left(1-p\right) P\;\left(Y=1\right)=\omega\cdot p,$$

where *ω* is the probability of exposure and *p* is the probability of occurrence of the phenomenon of interest among exposed individuals, as shown in Fig. 1. According to this scheme, the proportion of structural zeros will be 1 − *ω* and the proportion of non-structural zeros will be *ω* · (1 − *p*).

To overcome the impossibility of models without covariates based on the frequen- tist approach to differentiate between structural and non-structural zeros, in this work a model within the Bayesian framework is proposed. In this context, we as- sume that the prior distribution of the parameter of the first Bernoulli *ω* is uniform between 0 and 0.5 while the prior distribution of the probability of success in the second Bernoulli *p* is uniform between 0.5 and 1. In this way, the proposed model will be able to distinguish the two sources of zeros. Obviously, these hypotheses can be modified based on prior knowledge of the parameters that govern the phenomenon under investigation by making simple changes to the posterior distributions defined in Eq. (6) and recalculating the marginals shown in Eq. (5). This distinction is not necessary if covariates are included in the model since the covariates allow the origin of the zeros to be distinguished. To ensure that the estimates are kept within the appropriate parameter space, the *logit* link, commonly used in logistic regression, has been used.

3 $${\displaystyle \begin{array}{c}{\log} it\left(\omega \right)=\log \left(\frac{\omega }{1-\omega}\right)={\theta}_0+{\theta}_1{X}_1+\dots +{\theta}_k{X}_k\\ {}{\log} it(p)=\log \left(\frac{p}{1-p}\right)={\beta}_0+{\beta}_1{Z}_1+\dots {\beta}_m{Z}_{m,}\end{array}}$$

where *X*_1_*,*. *.., X*_*k*_ are the covariates that have a hypotetical impact over the zero inflated part and *Z*_1_*,. .., Z*_*m*_ are the covariates that might have an influence over the non zero inflated part. The parameters *θ*_*i*_, *i* = 0*,. .., k* and *β*_*j*_, *j* = 0*,. .., m* are assumed to follow a normal distribution with mean 0 and variance *σ*^2^ and ${\sigma}_{\theta}^2$ and ${\sigma}_{\beta}^2$ respectively, modeled as hyperparameters.

The models proposed to analyse the data described in the following section and in the simulation study have been written in the programming language *Stan*, within the *R* environment [14] and are freely available from the authors as a package called *bayesZIB* [15]. To the best of our knowledge, this is the only package available in R able to fit zero-inflated Bernoulli regression models. The use of the package is very similar to other packages that implement zero-inflated models, such as *pscl* [16], to facilitate the interpretation of the results, while more advanced users could easily adapt the code to their specific requirements. If necessary, appropriate priors for the parameters *ω* and *p* can be defined in the function bayesZIB using the argument priors (only uniforms with different parameters are implemented so far in the package).

### No covariates

In the particular case in which the interest is in estimating the proportion of struc- tural (1-*ω*) and sample (1-*p*) zeros without accounting for the effect of any covariate, the *posterior* distributions of *ω* and *p* can be obtained analytically assuming some a priori knowledge of their distributions. As mentioned before, one could set *ω* to be uniform distributed on [0*,* 0*.*5] and *p* to be uniform distributed on [0*.*5*,* 1]. Be- cause the observations are Bernoulli(*p* · *ω*) distributed, the likelihood function can be written as

4 $$L\sim {\left(p\cdot \omega \right)}^m\cdot {\left(1-p\cdot \omega \right)}^{n-m},$$

where *m* is the frequency of occurrence of the phenomenon of interest and *n* is the total number of observations. From here, the joint *posterior* could be obtained as

5 $${\displaystyle \begin{array}{l}f\left(p,\omega \right)\sim {\left(p\cdot \varpi \right)}^m\cdot {\left(1-p\cdot \omega \right)}^{n-m}\cdot \\ {}{U}_{\left[0,1/2\right]}\left(\omega \right)\cdot {\mathrm{U}}_{\left[1/2,1\right]}(p)\end{array}}$$

From here the *posterior* marginal distributions of the two parameters can be obtained as

6 $${\displaystyle \begin{array}{l}f\left(\omega \right)\sim {\omega}^m{\int}_{1/2}^1{p}^m\cdot {\left(1-p\cdot \omega \right)}^{n-m} d p\sim \\ {}\kern2.28em \frac{1}{\omega}\cdot {\int}_{\omega /2}^{\omega }{t}^m\cdot {\left(1-t\right)}^{n-m} d t\sim \\ {}\kern2.4em \frac{F\left(\omega, m+1,n-m+1\right)-F\left(\frac{\omega }{2},m+1,n-m+1\right)}{\omega}\\ {}f(p)\sim {p}^m{\int}_o^{1/2}{\omega}^m\cdot {\left(1-p\cdot \omega \right)}^{n-m} d\omega \sim \\ {}\kern2.28em \frac{1}{p}\cdot {\int}_o^{p/2}{t}^m\cdot {\left(1-t\right)}^{n-m} d t\sim \\ {}\kern2.4em \frac{F\left(\frac{p}{2},m+1,n-m+1\right)}{p}\end{array}}$$

where *F* is the beta distribution function with parameters *m* + 1 and *n* − *m* + 1, implemented in the *R* function *pbeta*.

The methods used to analyse the real data example in the following sections are in accordance with relevant guidelines and regulations, in particular with the Inter- national Labour Organization criteria, also used to define the target population in the European Working Conditions Survey [17] or the EU Labour Force Survey [18]. Participation in the considered study was voluntary and confidential, and informed consent was obtained from all subjects in order to be included. The data were anal- ysed anonymously and all procedures were approved by the Ethics Committee on Animal and Human Experimentation of the Autonomous University of Barcelona (CEEAH/3445).

## Results

This section presents the results of the analyses using the proposed methodology over a real data set and they are compared to the most common alternatives. The performance of the method is also studied by means of a comprehensive simulation study, with and without covariates.

### Real data

In the database used to exemplify the use of the proposed methodology, we have a total of *n* = 1564 workers. Among these, it is known that 946 (around 61%) were not at risk of being presenteeist because they were not ill on any day during the study period. These observations correspond to the concept of structural zeros (1 − *ω* = 0*.*61), and an estimate of their proportion can be obtained by using zero inflated models, even taking into account the values of the variables used as explanations in the regression model. The proportion of presenteeists among those exposed is *p* = 0*.*70. Globally, a total of *m* = 430 workers experienced the event of interest. In the following subsections CI is used as an abbreviation of the confidence interval for frequentist analyses, and CrI is used for credibility interval when referring to the proposed Bayesian model.

#### Including the whole population

**Fig. 2:**
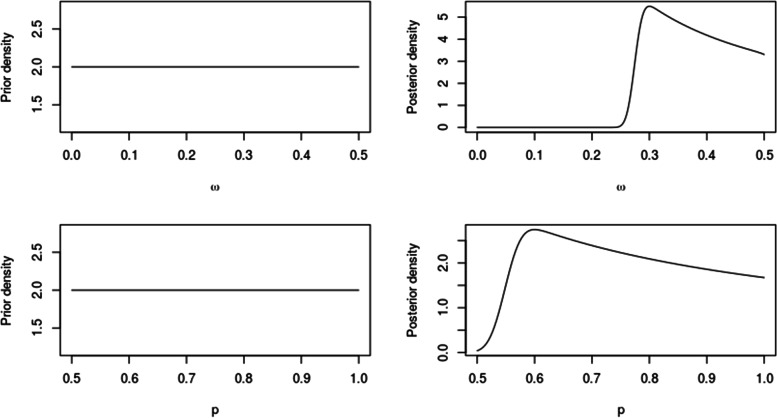
Prior (left column) and posterior (right column) distributions for *ω* and *p*

Taking all the population into consideration (*n* = 1564), i. e., including individuals at risk and not at risk (those who were not at risk during the study period), we fit a Bayesian zero inflated Bernoulli model, where the proportion of structural zeros 1 − *ω* is greater than 0.5 (prior uniform for *ω* at [0*,* 0*.*5]) and the proportion of sample zeros 1 − *p* is less than 0.5 (prior uniform for *p* at [0*.*5*,* 1]). This information is extracted from [19]. In this case, without using covariates, the model allows estimating the values of *ω*ˆ = 0*.*37 (95% CrI: 0.27–0.49) and *p*ˆ = 0*.*74 (95% CrI: 0.55–0.99). Here *ω*ˆ and *p*ˆ indicate the median of the marginal posterior of *ω* and *p* respectively. The a priori and a posteriori marginal distributions of both parameters are shown in Fig. 2. The different shapes between marginal priors and posteriors show that the models learn from the data.

On the other hand, analysing these data as is traditional in the literature, using a logistic regression model without taking into account that there are subjects who have not been at risk, the proportion obtained from presentists is 0.27 (95% CI: 0.25–0.30), a value with a controversial interpretation since it is significantly underestimating the proportion of presenters if the subjects who have not been at risk of being present are excluded, since it is ultimately an estimate of *ω*ˆ · *p*ˆ, being impossible to identify the two parameters.

Additionally, the proposed model allows incorporating covariates in both pro- cesses. To illustrate how it works, we will consider here the self-perceived general state of health (categorized as good or bad) and the feeling of being replaceable, which is an item included in the vulnerability dimension of the Employment Pre- cariousness Scale [20], with categories “Always”, “Sometimes” and “Never”. The hypothesis is that the general state of health would be related to the risk of be- ing present (zero inflated part of the model) and the feeling of being replaceable would be related to presenting the phenomenon once a worker is exposed (non zero inflated part in the model), so the model is including one covariate in each part (*k* = *m* = 1). The model was fitted using 5 Markov chains, 5000 iterations in each chain (half used for warmup and half for inference), a target average acceptance probability of 0.999 and a maximum allowed treedepth of 25. Notice that these technical values might need to be changed depending on the analysed data. As can be seen in the next section, the results of the model shown in Table 1 largely co- incide with the standard logistic analyses reported in Table 2, particularly in the direction and impact of associations. All *R* codes used in this paper are available as Supplementary Material.

**Table 1:** Bayesian analysis on whole population. CrI stands for credible interval

	Covariate	Coefficient. (95% CrI)
Struct	Intercept	−0.40 (− 0.83, 0.22)
	Bad	1.43 (0.83, 2.58)
Non-struct.	Intercept	1.03 (0.16, 2.32)
	Sometimes	0.03 (−0.73, 1.37)
	Never	−0.68 (− 1.38, − 0.15)

**Table 2:** Logistic regression on all population and only on exposed individuals. CI stands for confidence interval

Population	Covariate	Coefficient. (95% CI)
Whole population	Intercept	1.07 (− 1.19, − 0.95)
	Bad	1.05 (0.68, 1.42)
Only exposed	Intercept	1.16 (0.79, 1.53)
	Sometimes	−0.02 (− 0.60, 0.56)
	Never	− 0.54 (− 0.97, − 0.11)

Similarly to other regression models, the effect of never having the feeling of being replaceable over the odds of attending work when sick for someone who is at risk compared to workers who always have that sensation can be quantified by *e*^*−* 1*.*04^ = 0*.*35.

#### Excluding healthy population

If the information about which subjects are actually exposed to the phenomenon of interest is available (an ideal but unusual situation in practice), unexposed subjects could be excluded and a logistic regression model could be adjusted to the exposed individuals. Using the same explanatory variables described in the previous section, the corresponding coefficients and their 95% confidence intervals are described in Table 2.

### Simulation study

In order to check the performance of the proposed methodology, 100 random samples were generated for each considered sample size (*n* = 500*,* 1500), and combination of parameters. The zero inflated part was build upon the logistic regression model *logit*(*P* (*X* = 1)) = *θ*_0_ + *θ*_1_ · *x*_1_ + *θ*_2_ · *x*_2_, where *x*_1_ and *x*_2_ are two independent covariates, each following a standard normal distribution. The non zero inflated part was build upon the logistic regression model *logit*(*P* (*Y* = 1 | *X* = 1)) = *β*_0_ + *β*_1_ · *x*_3_ + *β*_2_ · *x*_4_, where *x*_3_ and *x*_4_ are two independent covariates, each with a standard normal distribution. To cover different effect magnitudes, the following values for each parameter were considered:

- *β*_0_ = 0*.*5*,* 1*,* 2
- *β*_1_ = 2*,* 3*,* 4
- *β*_2_ = 3
- *θ*_0_ = − 0*.*5*,* − 1*,* − 2
- *θ*_1_ = − 2*,* − 3*,* − 4
- *θ*_2_ = − 3

It is important to notice that this is an extreme situation, in which we consider that all the mass probability of the parameter distributions is concentrated in one point, the “true” value of the parameter.

For each random sample, the *posterior* marginal distributions of the parameters have been summarised by their median and percentiles 2.5 and 97.5%.

Tables 3 and 4 show, for each combination of parameters, the average estimates and upper and lower limits of the 95% credibility intervals. As no relevant differ- ences were observed regarding sample sizes, Tables 3 and 4 shows only the results corresponding to *n* = 1500. The results corresponding to *n* = 500 are available as Supplementary Material. It can be seen that in all cases the original parameters used to generate the simulations can be properly recovered by the fitted models.

**Table 3:** Simulation study results including covariates (I)

*β*_0_	*β*_1_	*θ*_0_	*θ*_1_	*β*_0_ (95% CrI)	*β*_1_ (95% CrI)	*β*_2_ (95% CrI)	*θ*_0_ (95% CrI)	*θ*_1_ (95% CrI)	*θ*_2_ (95% CrI)
			−2	0.5 (0.2, 0.9)	2 (1.6, 2.5)	3 (2.5, 3.7)	−0.5 (− 0.8, − 0.2)	− 2 (− 2.5, − 1.7)	− 3.1 (− 3.6, − 2.6)
		−0.5	− 3	0.5 (0.2, 0.9)	2 (1.6, 2.5)	3 (2.5, 3.7)	−0.5 (− 0.8, − 0.2)	− 3 (− 3.6, − 2.5)	− 3 (− 3.6, − 2.5)
			− 4	0.5 (0.2, 0.8)	2 (1.6, 2.5)	3 (2.5, 3.6)	− 0.5 (− 0.8, − 0.2)	− 3.9 (− 4.7, − 3.3)	− 2.9 (− 3.5, − 2.4)
			− 2	0.5 (0.2, 0.9)	2 (1.6, 2.5)	3 (2.4, 3.7)	− 1 (− 1.3, − 0.7)	− 2 (− 2.5, − 1.7)	−3.1 (− 3.6, − 2.6)
	2	− 1	− 3	0.5 (0.2, 0.9)	2 (1.6, 2.5)	3 (2.4, 3.7)	−1 (− 1.3, − 0.7)	−3 (− 3.6, − 2.5)	−3 (− 3.6, − 2.5)
			−4	0.5 (0.2, 0.9)	2 (1.6, 2.5)	3.1 (2.5, 3.7)	−1 (− 1.3, − 0.7)	−3.8 (− 4.6, − 3.2)	− 2.9 (− 3.5, − 2.4)
			− 2	0.5 (0.1, 1)	2 (1.5, 2.6)	3 (2.3, 3.8)	− 2 (− 2.4, − 1.7)	−2 (− 2.5, − 1.6)	−3.1 (− 3.6, − 2.6)
		−2	− 3	0.5 (0.1, 0.9)	2 (1.5, 2.6)	3 (2.4, 3.8)	−2 (− 2.3, − 1.6)	− 3 (− 3.6, − 2.4)	− 3 (− 3.6, − 2.5)
			−4	0.5 (0.2, 0.9)	2 (1.5, 2.5)	3 (2.4, 3.7)	−1.9 (− 2.3, − 1.6)	− 3.9 (− 4.7, − 3.3)	− 2.9 (− 3.5, − 2.4)
			− 2	0.5 (0.2, 0.9)	3 (2.4, 3.7)	3 (2.4, 3.7)	−0.5 (− 0.8, − 0.2)	− 2 (− 2.4, − 1.7)	− 3 (− 3.6, − 2.6)
		−0.5	− 3	0.5 (0.2, 0.9)	3 (2.5, 3.7)	3 (2.4, 3.6)	−0.5 (− 0.8, − 0.2)	− 3 (− 3.6, − 2.5)	− 3 (− 3.6, − 2.5)
			−4	0.5 (0.2, 0.8)	3 (2.5, 3.6)	3 (2.4, 3.6)	− 0.5 (− 0.8, − 0.2)	− 3.9 (− 4.7, − 3.3)	− 2.9 (− 3.5, − 2.4)
			− 2	0.5 (0.1, 0.9)	2.9 (2.3, 3.7)	2.9 (2.3, 3.7)	−1 (− 1.2, − 0.7)	− 2 (− 2.4, − 1.6)	− 3 (− 3.5, − 2.5)
0.5	3	−1	− 3	0.5 (0.2, 0.9)	2.9 (2.4, 3.6)	3 (2.4, 3.6)	−1 (− 1.3, − 0.7)	− 3 (− 3.6, − 2.5)	− 3 (− 3.6, − 2.5)
			−4	0.5 (0.2, 0.8)	3 (2.4, 3.6)	3 (2.4, 3.6)	−1 (− 1.3, − 0.7)	−3.9 (− 4.7, − 3.3)	− 2.9 (− 3.5, − 2.4)
			− 2	0.4 (0, 0.9)	2.9 (2.2, 3.7)	2.9 (2.2, 3.7)	−2 (− 2.4, − 1.7)	−2 (− 2.4, − 1.6)	− 3 (− 3.6, − 2.5)
		−2	−3	0.5 (0.1, 1)	3 (2.3, 3.8)	3 (2.3, 3.8)	−2 (− 2.4, − 1.6)	− 3 (− 3.6, − 2.5)	− 3 (− 3.6, − 2.5)
			− 4	0.5 (0.1, 0.9)	3 (2.4, 3.7)	3 (2.4, 3.7)	−2 (− 2.4, − 1.6)	− 3.9 (− 4.7, − 3.3)	−2.9 (− 3.5, − 2.4)
			− 2	0.4 (0.1, 0.8)	3.9 (3.2, 4.8)	2.9 (2.4, 3.6)	−0.5 (− 0.7, − 0.2)	− 2 (− 2.4, − 1.6)	− 3 (− 3.5, − 2.5)
		−0.5	− 3	0.5 (0.1, 0.8)	3.9 (3.2, 4.7)	2.9 (2.3, 3.6)	−0.5 (− 0.8, − 0.2)	− 3 (− 3.5, − 2.5)	− 3 (− 3.5, − 2.5)
			−4	0.5 (0.2, 0.9)	4 (3.2, 4.8)	3 (2.4, 3.6)	− 0.5 (− 0.8, − 0.2)	− 3.9 (− 4.6, − 3.3)	−2.9 (− 3.5, − 2.4)
			− 2	0.5 (0.1, 0.9)	3.8 (3.1, 4.8)	2.9 (2.3, 3.6)	−1 (− 1.3, − 0.7)	− 2 (− 2.4, − 1.6)	− 3 (− 3.5, − 2.5)
	4	−1	− 3	0.5 (0.1, 0.9)	3.9 (3.1, 4.8)	2.9 (2.3, 3.6)	−1 (− 1.3, − 0.7)	−3 (− 3.5, − 2.5)	−3 (− 3.5, − 2.5)
			−4	0.5 (0.1, 0.9)	3.9 (3.2, 4.8)	2.9 (2.4, 3.6)	− 1 (− 1.3, − 0.7)	− 4 (− 4.7, − 3.3)	− 3 (− 3.6, − 2.5)
			−2	0.4 (0, 0.9)	3.8 (2.9, 4.9)	2.9 (2.2, 3.7)	−2 (− 2.4, − 1.7)	−2 (− 2.5, − 1.7)	−3 (− 3.6, − 2.5)
		−2	− 3	0.5 (0.1, 0.9)	3.9 (3.1, 5)	2.9 (2.3, 3.7)	−2 (− 2.4, − 1.6)	− 3 (− 3.6, − 2.5)	− 3 (− 3.6, − 2.5)
			−4	0.5 (0.1, 0.9)	3.9 (3.1, 4.8)	2.9 (2.3, 3.7)	−2 (− 2.4, − 1.6)	− 3.9 (− 4.7, − 3.3)	−2.9 (− 3.5, − 2.4)
			− 2	1 (0.7, 1.5)	2 (1.6, 2.5)	3 (2.5, 3.7)	−0.5 (− 0.8, − 0.3)	−2 (− 2.4, − 1.7)	− 3 (− 3.6, − 2.6)
		−0.5	− 3	1 (0.7, 1.4)	2 (1.6, 2.4)	3 (2.5, 3.6)	−0.5 (− 0.7, − 0.2)	− 3 (− 3.6, − 2.5)	− 3 (− 3.5, − 2.5)
			−4	1 (0.7, 1.4)	2 (1.6, 2.5)	3 (2.5, 3.6)	− 0.5 (− 0.8, − 0.2)	− 3.9 (− 4.6, − 3.3)	−2.9 (− 3.5, − 2.4)
			− 2	1 (0.6, 1.4)	2 (1.5, 2.5)	3 (2.4, 3.7)	−1 (− 1.2, − 0.7)	−2 (− 2.4, − 1.7)	− 3 (− 3.6, − 2.6)
	2	−1	− 3	1 (0.6, 1.4)	2 (1.6, 2.5)	3 (2.4, 3.6)	−1 (− 1.3, − 0.7)	− 3 (− 3.6, − 2.5)	− 3 (− 3.6, − 2.5)
			−4	1 (0.7, 1.4)	2 (1.6, 2.5)	3 (2.5, 3.7)	−1 (− 1.3, − 0.7)	− 3.9 (− 4.6, − 3.3)	− 2.9 (− 3.5, − 2.4)
			− 2	1 (0.5, 1.6)	2 (1.4, 2.6)	3 (2.3, 3.8)	−2 (− 2.3, − 1.7)	−2 (− 2.4, − 1.6)	− 3 (− 3.6, − 2.5)
		−2	− 3	1 (0.6, 1.5)	2 (1.5, 2.6)	3 (2.4, 3.8)	−2 (− 2.4, − 1.7)	− 3 (− 3.5, − 2.5)	− 3 (− 3.6, − 2.5)
			− 4	1 (0.6, 1.5)	2 (1.5, 2.5)	3 (2.4, 3.7)	−1.9 (− 2.3, − 1.6)	− 3.9 (− 4.6, − 3.3)	− 2.9 (− 3.5, − 2.4)
			− 2	1 (0.6, 1.4)	2.9 (2.4, 3.6)	2.9 (2.3, 3.6)	−0.5 (− 0.7, − 0.2)	−2 (− 2.4, − 1.7)	−3.1 (− 3.6, − 2.6)
		−0.5	− 3	1 (0.6, 1.4)	3 (2.4, 3.6)	3 (2.4, 3.6)	−0.5 (− 0.8, − 0.2)	− 3 (− 3.5, − 2.5)	−3 (− 3.5, − 2.5)
			− 4	1 (0.7, 1.4)	3 (2.5, 3.6)	3 (2.4, 3.6)	− 0.5 (− 0.8, − 0.2)	− 4 (− 4.7, − 3.4)	− 3 (− 3.6, − 2.5)
			− 2	0.9 (0.5, 1.4)	2.9 (2.3, 3.6)	3 (2.4, 3.7)	−1 (− 1.2, − 0.7)	− 2 (− 2.4, − 1.7)	− 3 (− 3.6, − 2.6)
1	3	−1	−3	1 (0.6, 1.4)	3 (2.4, 3.7)	3 (2.4, 3.7)	−1 (− 1.3, − 0.7)	− 3 (− 3.6, − 2.5)	− 3 (− 3.5, − 2.5)
			− 4	1 (0.6, 1.4)	3 (2.4, 3.6)	3 (2.4, 3.6)	−1 (− 1.3, − 0.7)	−3.9 (− 4.6, − 3.3)	− 2.9 (− 3.5, − 2.4)
			−2	1 (0.5, 1.6)	3 (2.3, 3.9)	2.9 (2.3, 3.8)	−2 (− 2.4, − 1.7)	−2 (− 2.4, − 1.7)	− 3 (− 3.6, − 2.6)
		−2	−3	0.9 (0.5, 1.5)	2.9 (2.3, 3.7)	2.9 (2.3, 3.7)	−2 (− 2.3, − 1.6)	− 3 (− 3.6, − 2.5)	− 3 (− 3.6, − 2.5)
			− 4	0.9 (0.5, 1.4)	2.9 (2.3, 3.7)	2.9 (2.3, 3.7)	−1.9 (− 2.3, − 1.6)	−3.9 (− 4.7, − 3.3)	− 2.9 (− 3.5, − 2.4)
			− 2	0.9 (0.6, 1.4)	3.9 (3.1, 4.7)	2.9 (2.3, 3.6)	−0.5 (− 0.7, − 0.3)	− 2 (− 2.4, − 1.7)	−3 (− 3.5, − 2.6)
		−0.5	− 3	1 (0.6, 1.4)	3.9 (3.2, 4.8)	2.9 (2.4, 3.6)	−0.5 (− 0.8, − 0.2)	− 3 (− 3.6, − 2.5)	−3 (− 3.6, − 2.6)
			−4	0.9 (0.6, 1.3)	3.9 (3.2, 4.7)	2.9 (2.4, 3.6)	− 0.5 (− 0.7, − 0.2)	− 4 (− 4.7, − 3.4)	− 3 (− 3.5, − 2.5)
			− 2	0.9 (0.5, 1.4)	3.9 (3.1, 4.8)	2.8 (2.2, 3.6)	−1 (− 1.3, − 0.7)	− 2 (− 2.4, − 1.7)	−3 (− 3.6, − 2.6)
	4	−1	−3	1 (0.6, 1.4)	3.9 (3.2, 4.8)	2.9 (2.4, 3.7)	−1 (− 1.2, − 0.7)	− 3 (− 3.6, − 2.5)	− 3 (− 3.6, − 2.5)
			−4	1 (0.6, 1.4)	3.9 (3.2, 4.7)	2.9 (2.3, 3.6)	−0.9 (− 1.2, − 0.7)	−4 (− 4.7, − 3.3)	−3 (− 3.5, − 2.5)
			− 2	0.9 (0.4, 1.5)	3.8 (2.9, 4.9)	2.9 (2.2, 3.7)	−2 (− 2.3, − 1.7)	−2 (− 2.4, − 1.7)	− 3 (− 3.5, − 2.5)
		−2	−3	1 (0.5, 1.5)	3.9 (3, 4.9)	2.9 (2.2, 3.7)	−2 (− 2.4, − 1.7)	−3 (− 3.5, − 2.5)	−3 (− 3.6, − 2.5)
			−4	0.9 (0.5, 1.4)	3.9 (3.1, 4.9)	2.9 (2.3, 3.7)	−1.9 (− 2.3, − 1.6)	− 3.9 (− 4.6, − 3.3)	− 3 (− 3.5, − 2.5)

**Table 4:** Simulation study results including covariates (II)

*β*_0_	*β*_1_	*θ*_0_	*θ*_1_	*β*_0_ (95% CrI)	*β*_1_ (95% CrI)	*β*_*2*_ (95% CrI)	*θ*_0_ (95% CrI)	*θ*_1_ (95% CrI)	*θ*_2_ (95% CrI)
			−2	1.9 (1.5, 2.5)	2 (1.5, 2.5)	3 (2.4, 3.7)	−0.5 (− 0.7, − 0.2)	−2 (− 2.4, − 1.7)	−3.1 (− 3.6, − 2.6)
		−0.5	−3	2 (1.6, 2.6)	2 (1.6, 2.5)	3 (2.4, 3.6)	−0.5 (− 0.8, − 0.3)	−3 (− 3.6, − 2.6)	−3 (− 3.5, − 2.6)
			−4	2 (1.6, 2.5)	2 (1.6, 2.5)	3 (2.4, 3.6)	− 0.5 (− 0.8, − 0.2)	−3.9 (− 4.6, − 3.4)	−3 (− 3.5, − 2.5)
			−2	1.9 (1.4, 2.5)	1.9 (1.5, 2.5)	2.9 (2.3, 3.6)	−1 (− 1.2, − 0.8)	− 2 (− 2.4, − 1.7)	− 3.1 (− 3.6, − 2.6)
	2	−1	−3	2 (1.5, 2.6)	1.9 (1.5, 2.5)	2.9 (2.4, 3.6)	−1 (− 1.2, − 0.7)	− 3 (− 3.5, − 2.6)	−3 (− 3.5, − 2.5)
			−4	2 (1.6, 2.6)	2 (1.6, 2.5)	3 (2.4, 3.7)	−1 (− 1.3, − 0.7)	− 4 (− 4.7, − 3.4)	− 3 (− 3.5, − 2.5)
			−2	1.9 (1.3, 2.7)	2 (1.5, 2.7)	2.9 (2.2, 3.8)	−2 (− 2.3, − 1.7)	−2 (− 2.4, − 1.7)	−3 (− 3.5, − 2.6)
		−2	−3	2 (1.4, 2.7)	2 (1.5, 2.6)	2.9 (2.3, 3.8)	−2 (− 2.3, − 1.7)	−3 (− 3.5, − 2.5)	−3 (− 3.5, − 2.5)
			− 4	1.9 (1.4, 2.6)	1.9 (1.5, 2.5)	2.9 (2.3, 3.7)	−2 (− 2.3, − 1.7)	−4 (− 4.7, − 3.4)	− 3 (− 3.5, − 2.5)
			−2	2 (1.5, 2.6)	3 (2.4, 3.7)	3 (2.4, 3.7)	−0.5 (− 0.7, − 0.2)	−2 (− 2.4, − 1.7)	−3 (− 3.5, − 2.6)
		−0.5	−3	2 (1.5, 2.5)	3 (2.4, 3.6)	2.9 (2.4, 3.6)	−0.5 (− 0.7, − 0.3)	−3 (− 3.5, − 2.6)	−3 (− 3.5, − 2.5)
			−4	1.9 (1.5, 2.5)	3 (2.4, 3.6)	2.9 (2.4, 3.6)	−0.5 (− 0.7, − 0.2)	−3.9 (− 4.6, − 3.4)	− 2.9 (− 3.5, − 2.5)
			−2	1.9 (1.4, 2.6)	2.9 (2.3, 3.7)	2.9 (2.3, 3.7)	−1 (− 1.3, − 0.8)	− 2 (− 2.4, − 1.7)	−3 (− 3.5, − 2.6)
2	3	−1	−3	1.9 (1.4, 2.5)	2.9 (2.3, 3.6)	2.9 (2.3, 3.6)	−1 (− 1.3, − 0.7)	−3 (− 3.5, − 2.5)	−3 (− 3.5, − 2.6)
			− 4	1.9 (1.5, 2.5)	2.9 (2.3, 3.6)	2.9 (2.4, 3.6)	−1 (− 1.3, − 0.7)	−4 (− 4.7, − 3.4)	− 3 (− 3.6, − 2.5)
			−2	1.9 (1.3, 2.6)	2.9 (2.2, 3.7)	2.9 (2.2, 3.8)	−2 (− 2.3, − 1.7)	−2 (− 2.4, − 1.7)	−3 (− 3.5, − 2.6)
		−2	− 3	1.9 (1.3, 2.6)	2.9 (2.3, 3.7)	2.9 (2.2, 3.7)	−2 (− 2.3, − 1.7)	−3 (− 3.5, − 2.6)	−3 (− 3.5, − 2.5)
			− 4	2 (1.4, 2.6)	2.9 (2.3, 3.7)	2.9 (2.3, 3.7)	−1.9 (− 2.3, − 1.6)	− 3.9 (− 4.5, − 3.3)	−2.9 (− 3.4, − 2.5)
			− 2	1.9 (1.4, 2.5)	3.8 (3.1, 4.7)	2.9 (2.3, 3.6)	−0.5 (− 0.7, − 0.3)	−2 (− 2.4, − 1.7)	−3.1 (− 3.5, − 2.6)
		−0.5	−3	1.9 (1.4, 2.5)	3.8 (3.1, 4.7)	2.9 (2.3, 3.6)	−0.5 (− 0.7, − 0.2)	−3 (− 3.6, − 2.6)	−3 (− 3.6, − 2.6)
			−4	1.9 (1.4, 2.4)	3.8 (3.1, 4.7)	2.9 (2.3, 3.6)	−0.5 (− 0.7, − 0.2)	−4 (− 4.7, − 3.4)	−3 (− 3.5, − 2.5)
			−2	1.9 (1.4, 2.6)	3.8 (3, 4.8)	2.9 (2.3, 3.7)	−1 (− 1.2, − 0.8)	− 2 (− 2.4, − 1.7)	− 3 (− 3.5, − 2.6)
	4	−1	−3	1.9 (1.4, 2.5)	3.9 (3.1, 4.8)	2.9 (2.3, 3.6)	−1 (− 1.3, − 0.7)	− 3 (− 3.5, − 2.6)	−3 (− 3.5, − 2.6)
			− 4	2 (1.5, 2.5)	3.9 (3.2, 4.8)	2.9 (2.3, 3.6)	− 1 (− 1.2, − 0.7)	−3.9 (− 4.6, − 3.4)	−3 (− 3.5, − 2.5)
			−2	1.8 (1.2, 2.6)	3.8 (2.9, 4.9)	2.8 (2.1, 3.7)	−2 (− 2.3, − 1.7)	−2 (− 2.4, − 1.7)	−3 (− 3.5, − 2.6)
		−2	− 3	1.8 (1.3, 2.5)	3.7 (2.9, 4.7)	2.8 (2.1, 3.6)	−2 (− 2.3, − 1.7)	− 3.1 (− 3.6, − 2.6)	−3 (− 3.6, − 2.6)
			−4	1.9 (1.4, 2.6)	3.8 (3, 4.8)	2.9 (2.2, 3.7)	−1.9 (− 2.3, − 1.6)	− 3.9 (− 4.6, − 3.3)	−2.9 (− 3.5, − 2.5)

The *R* code used for the simulation is available as Supplementary Material. An additional simulation was conducted to evaluate the performance of the proposed methodology when there are no covariates involved, the details and results of this simulation can also be find in the Supplementary Material (Appendix A, Table S2).

## Discussion

The proposed methodology is able to distinguish two different sources of zeros (structural and non-structural) from dichotomous data in a Bayesian framework by assuming priors with different parameters on proportion of structural and non- structural zeros. Furthermore, since it is freely available as an *R* package, it is easily usable for any researcher who needs to adjust this type of data and easily modifiable for more advanced users who need to adapt the model to their context, for example with different choices of the *prior* distributions of *ω* and *p*.

The approach used to analyse the SP is an important topic. Some studies include all working population to estimate SP, whilst other exclude “healthy” workers. As result, different conclusions in terms of prevalence and associated factors are obtained [21]. SP is an outcome resulting from mixing two phenomena, i.e. health status and exercise of rights. Health status plays a role regarding the fact of being exposed; and, among the exposed, the lack of the exercise of the right to take a sick leave determines SP. Using the proposed ZIB approach one could describe, in a single analysis, both phenomena: first, which factors are associated to the exposure to presenteeism (to be “sick”, factors related to health status), and after that, which factors increase the probability of being presenteeist among the exposed workers.

The simulation study shows that, even with relatively small sample sizes the model is capable of producing reasonable estimates for the parameters involved in both the zero inflated and non zero inflated processes. As expected, the credibility intervals length diminishes with sample size while their coverage grows.

## Conclusions

The proposed method is a reliable alternative for the analysis of zero inflated di- chotomous outcomes, as shown by the simulation study, and can be very useful in situations when there are two potential and indistinguishable sources of zeros. If there are covariates to be included in the model, the method is able to use them in order to identify the subpopulation at risk, and the Bayesian strategy assures that the two sources of zeros may be detected even when there are no covariates by utilizing different priors for the probability of success of each Bernoulli variable. The proposed model has been compiled in the form of the *bayesZIB* R package [15], so it is publicly available for any researcher facing this issue.

## Supplementary Information

**Additional file 1.** Additional tables and code. Additional tables obtained from the simulation study and R code to reproduce the analyses.

## Data Availability

The dataset analysed during the current study is available in the GitHub repository, https://github.com/dmorinya/BayesZIB. R codes used to analyse the real data and to generate the data used in the simulation study are available as supplementary material.

## Abbreviations

CI: Confidence interval
CrI: Credible interval
EM: Expectation-Maximization
SP: Sickness presenteeism
ZIB: Zero inflated Bernoulli
ZINB: Zero inflated negative binomial
ZIP: Zero inflated Poisson
