# Correction: Analysis of zero inflated dichotomous variables from a Bayesian perspective: application to occupational health

**DOI:** 10.1186/s12874-022-01697-4

**Published:** 2022-08-04

**Authors:** David Moriña, Pedro Puig, Albert Navarro

**Affiliations:** 1 Department of Econometrics, Statistics and Applied Econometrics, Riskcenter-IREA, Universitat de Barcelona (UB), Avinguda Diagonal 690, 08034 Barcelona, Spain; 2 Centre de Recerca Matemàtica, Universitat Autònoma de Barcelona (UAB), 08193 Cerdanyola del Vallès, Spain; 3 Departament de Matemàtiques, Universitat Autònoma de Barcelona (UAB), Edifci C, Campus de Bellaterra, 08193 Cerdanyola del Vall̀es, Spain; 4 Research group on Psychosocial risks, Organization of Work and Health (POWAH), Universitat Autònoma de Barcelona (UAB), Cerdanyola del Vallès, Spain; 5 Unitat de Bioestadística, Facultat de Medicina, Universitat Autònoma de Barcelona, Cerdanyola del Vallès, Spain

**Correction: BMC Med Res Methodol 21, 277 (2021)**

**https://doi.org/10.1186/s12874-021-01427-2**

Following publication of the original article [1], the authors noticed the incorrect affiliation 4 “Universitat Autònoma de Barcelona (UAB), Cerdanyola del Vallès, Spain.” in the published online version of this article. It should be corrected as “Research group on Psychosocial risks, Organization of Work and Health (POWAH), Universitat Autònoma de Barcelona (UAB), Cerdanyola del Vallès, Spain.”. The original article has been updated.
