# Supplementary material for: Analysis of zero inflated dichotomous variables from a Bayesian perspective: application to occupational health
Source: BMC Med Res Methodol. 2021 Dec 12;21:277. doi: 10.1186/s12874-021-01427-2 (PMC8667382; doi:10.1186/s12874-021-01427-2)
Supplement: Supplementary file 1 — Additional file 1. Additional tables and code. Additional tables obtained from the simulation study and R code to reproduce the analyses. [file 12874_2021_1427_MOESM1_ESM.zip › Supplementary material.pdf]

SUPPLEMENTARY MATERIAL

Accompanying the manuscript:

Analysis of zero inflated dichotomous variables from a Bayesian perspective: Application to occupational health

David Moriña
Department of Econometrics, Statistics and Applied Economics, Riskcenter-IREA, Universitat de Barcelona (UB), Barcelona, Spain
Centre de Recerca Matemàtica (CRM)
E-mail: dmorina@ub.edu

Pedro Puig
Barcelona Graduate School of Mathematics (BGSMath), Departament de Matemàtiques, Universitat Autònoma de Barcelona
Centre de Recerca Matemàtica (CRM)

Albert Navarro
Group on Psychosocial risks, Organization of Work and Health (POWAH), Universitat Autònoma de Barcelona
Unitat de Bioestadística, Facultat de Medicina, Universitat Autònoma de Barcelona

1. Supplementary tables

Table S1. Simulation study results including covariates (n=500).

β_0	β_1	θ_0	θ_1	$\widehat{\beta}_0$ (95% CrI)	$\widehat{\beta}_1$ (95% CrI)	$\widehat{\beta}_2$ (95% CrI)	$\widehat{\theta}_0$ (95% CrI)	$\widehat{\theta}_1$ (95% CrI)	$\widehat{\theta}_2$ (95% CrI)
0.5	2	-0.5	-2	0.5 (0, 1.2)	2 (1.3, 2.9)	3 (2.1, 4.1)	-0.5 (-1, 0)	-2 (-2.8, -1.4)	-3 (-4, -2.2)
			-3	0.5 (0, 1.2)	2 (1.4, 2.8)	3 (2.1, 4)	-0.4 (-0.9, 0)	-3 (-4, -2.2)	-2.9 (-3.9, -2.1)
			-4	0.5 (0, 1.1)	2 (1.4, 2.8)	3 (2.2, 4.1)	-0.5 (-1, 0.1)	-3.8 (-5, -2.8)	-2.8 (-3.8, -2)
		-1	-2	0.5 (-0.1, 1.2)	1.9 (1.3, 2.9)	2.9 (2, 4.1)	-1 (-1.5, -0.5)	-2 (-2.8, -1.4)	-3.1 (-4.1, -2.3)
			-3	0.5 (-0.1, 1.2)	2 (1.3, 2.9)	3 (2.1, 4.2)	-0.9 (-1.4, -0.4)	-3 (-4, -2.2)	-2.9 (-3.9, -2.1)
			-4	0.5 (0, 1.1)	2 (1.3, 2.8)	3 (2.1, 4.1)	-0.9 (-1.4, -0.4)	-3.7 (-4.9, -2.7)	-2.7 (-3.7, -1.9)
		-2	-2	0.5 (-0.2, 1.4)	2 (1.2, 3)	2.9 (1.9, 4.2)	-2 (-2.6, -1.4)	-1.9 (-2.7, -1.3)	-3 (-4, -2.2)
			-3	0.5 (-0.1, 1.3)	2 (1.3, 3)	3.1 (2.1, 4.4)	-1.9 (-2.6, -1.3)	-2.9 (-3.9, -2.1)	-2.9 (-3.9, -2.1)
			-4	0.5 (-0.1, 1.3)	2.1 (1.3, 3.1)	3.1 (2.1, 4.4)	-1.9 (-2.6, -1.3)	-3.8 (-5, -2.8)	-2.8 (-3.8, -2)
	3	-0.5	-2	0.5 (-0.1, 1.1)	2.9 (2, 4)	2.8 (2, 4)	-0.5 (-0.9, 0)	-2 (-2.7, -1.4)	-3 (-4, -2.2)
			-3	0.5 (0, 1.2)	3 (2.1, 4.1)	3 (2.1, 4.1)	-0.5 (-1, 0)	-3 (-4, -2.2)	-3 (-4, -2.2)
			-4	0.5 (0, 1.1)	2.9 (2.1, 4)	2.9 (2.1, 4)	-0.5 (-1, 0)	-3.8 (-5, -2.8)	-2.9 (-3.9, -2.1)
		-1	-2	0.4 (-0.1, 1.2)	2.9 (2, 4.1)	2.9 (2, 4.1)	-0.9 (-1.4, -0.5)	-2 (-2.8, -1.4)	-3 (-4, -2.3)
			-3	0.5 (-0.1, 1.2)	2.9 (2, 4)	2.9 (2, 4.1)	-1 (-1.5, -0.5)	-2.9 (-3.9, -2.1)	-2.9 (-3.9, -2.1)
			-4	0.5 (0, 1.2)	3 (2.1, 4.1)	3 (2.1, 4.1)	-1 (-1.5, -0.5)	-3.7 (-4.9, -2.7)	-2.8 (-3.8, -2)
		-2	-2	0.4 (-0.2, 1.3)	2.8 (1.8, 4.1)	2.8 (1.8, 4.2)	-2 (-2.6, -1.4)	-2 (-2.8, -1.4)	-3 (-4, -2.2)
			-3	0.4 (-0.2, 1.2)	2.8 (1.9, 4)	2.8 (1.8, 4)	-1.9 (-2.6, -1.3)	-2.9 (-3.9, -2.1)	-2.9 (-3.9, -2.1)
			-4	0.5 (-0.1, 1.2)	3 (2, 4.2)	3 (2, 4.2)	-1.9 (-2.6, -1.3)	-3.8 (-5, -2.8)	-2.8 (-3.8, -2)
	4	-0.5	-2	0.4 (-0.1, 1.1)	3.7 (2.6, 5.1)	2.7 (1.9, 3.8)	-0.5 (-0.9, 0)	-2 (-2.8, -1.4)	-3 (-4, -2.3)
			-3	0.5 (-0.1, 1.1)	3.7 (2.7, 5.1)	2.8 (1.9, 3.8)	-0.5 (-0.9, 0)	-3 (-3.9, -2.2)	-3 (-4, -2.2)
			-4	0.5 (-0.1, 1.1)	3.8 (2.7, 5.1)	2.8 (2, 3.9)	-0.5 (-1, 0)	-3.8 (-5.1, -2.9)	-2.9 (-3.9, -2.1)
		-1	-2	0.4 (-0.2, 1.1)	3.6 (2.5, 5)	2.7 (1.8, 3.9)	-1 (-1.5, -0.5)	-2.1 (-2.8, -1.5)	-3 (-4, -2.3)
			-3	0.4 (-0.1, 1.1)	3.6 (2.6, 5)	2.8 (1.9, 3.9)	-0.9 (-1.4, -0.4)	-2.9 (-3.9, -2.1)	-3 (-4, -2.2)
			-4	0.4 (-0.1, 1.1)	3.7 (2.6, 5)	2.8 (1.9, 3.9)	-1 (-1.5, -0.5)	-3.8 (-5.1, -2.9)	-2.9 (-3.9, -2.1)
		-2	-2	0.4 (-0.3, 1.2)	3.6 (2.4, 5.2)	2.6 (1.7, 3.9)	-1.9 (-2.5, -1.4)	-2 (-2.7, -1.4)	-3 (-4, -2.2)
			-3	0.4 (-0.3, 1.2)	3.7 (2.5, 5.2)	2.8 (1.8, 4)	-1.9 (-2.5, -1.3)	-2.9 (-3.8, -2.1)	-2.9 (-3.8, -2.1)

			-4	0.4 (-0.2, 1.2)	3.7 (2.6, 5.2)	2.8 (1.9, 4)	-1.9 (-2.6, -1.3)	-3.8 (-5, -2.8)	-2.9 (-3.9, -2.1)
			-2	1 (0.4, 1.7)	1.9 (1.3, 2.8)	3 (2.1, 4.1)	-0.5 (-0.9, 0)	-2 (-2.7, -1.4)	-3 (-4, -2.3)
		-0.5	-3	1 (0.4, 1.7)	2 (1.4, 2.9)	3 (2.1, 4)	-0.5 (-0.9, 0)	-3 (-4, -2.2)	-3 (-4, -2.2)
			-4	1.1 (0.5, 1.7)	2.1 (1.4, 2.9)	3.1 (2.2, 4.1)	-0.5 (-0.9, 0)	-3.8 (-5, -2.9)	-2.8 (-3.8, -2.1)
			-2	0.9 (0.3, 1.7)	1.9 (1.2, 2.8)	2.9 (2, 4.1)	-0.9 (-1.4, -0.5)	-2 (-2.7, -1.4)	-3.1 (-4, -2.3)
	2	-1	-3	1 (0.4, 1.8)	2 (1.3, 2.9)	2.9 (2.1, 4.1)	-1 (-1.4, -0.5)	-3 (-4, -2.2)	-3 (-4, -2.2)
			-4	1 (0.4, 1.7)	2 (1.3, 2.8)	3 (2.1, 4.1)	-0.9 (-1.4, -0.5)	-3.7 (-4.9, -2.8)	-2.8 (-3.7, -2)
			-2	1 (0.2, 1.9)	2 (1.1, 3)	2.9 (1.9, 4.2)	-1.9 (-2.5, -1.4)	-2 (-2.7, -1.4)	-3 (-4, -2.3)
		-2	-3	0.9 (0.3, 1.8)	2 (1.2, 2.9)	3 (2, 4.3)	-1.9 (-2.6, -1.4)	-2.9 (-3.9, -2.2)	-2.9 (-3.9, -2.1)
			-4	1 (0.3, 1.7)	2 (1.3, 3)	2.9 (2, 4.2)	-1.9 (-2.5, -1.3)	-3.8 (-5, -2.8)	-2.8 (-3.7, -2)
			-2	0.9 (0.3, 1.7)	2.9 (2, 4)	2.9 (2, 4)	-0.5 (-0.9, 0)	-2 (-2.7, -1.5)	-3.1 (-4, -2.3)
		-0.5	-3	1 (0.4, 1.7)	2.9 (2, 4)	2.8 (2, 3.9)	-0.5 (-0.9, 0)	-2.9 (-3.8, -2.2)	-2.9 (-3.8, -2.2)
			-4	0.9 (0.4, 1.6)	2.9 (2.1, 3.9)	2.9 (2, 3.9)	-0.4 (-0.9, 0)	-3.9 (-5.1, -2.9)	-2.9 (-3.9, -2.1)
			-2	0.9 (0.3, 1.7)	2.8 (1.9, 3.9)	2.8 (1.9, 4)	-0.9 (-1.4, -0.5)	-2 (-2.7, -1.4)	-3 (-4, -2.3)
1	3	-1	-3	1 (0.4, 1.8)	2.9 (2, 4.1)	2.9 (2, 4.1)	-1 (-1.5, -0.5)	-3 (-4, -2.2)	-3 (-4, -2.2)
			-4	0.9 (0.4, 1.7)	2.9 (2, 4)	2.9 (2, 4)	-0.9 (-1.4, -0.4)	-3.8 (-5, -2.9)	-2.9 (-3.8, -2.1)
			-2	0.9 (0.2, 1.9)	2.9 (1.8, 4.3)	2.8 (1.8, 4.2)	-2 (-2.6, -1.4)	-2 (-2.7, -1.4)	-3 (-3.9, -2.2)
		-2	-3	0.9 (0.2, 1.8)	2.9 (1.9, 4.2)	2.9 (1.9, 4.2)	-1.9 (-2.6, -1.4)	-2.9 (-3.9, -2.2)	-2.9 (-3.9, -2.1)
			-4	0.9 (0.2, 1.7)	2.9 (1.9, 4.1)	2.9 (1.9, 4.1)	-1.9 (-2.6, -1.3)	-3.8 (-5, -2.8)	-2.9 (-3.8, -2.1)
			-2	0.9 (0.3, 1.6)	3.6 (2.6, 5)	2.7 (1.9, 3.8)	-0.5 (-0.9, 0)	-2.1 (-2.8, -1.5)	-3.1 (-4.1, -2.4)
		-0.5	-3	0.9 (0.3, 1.6)	3.7 (2.6, 5)	2.8 (1.9, 3.9)	-0.5 (-0.9, 0)	-3 (-4, -2.2)	-3 (-4, -2.3)
			-4	0.9 (0.4, 1.6)	3.7 (2.7, 5)	2.7 (1.9, 3.8)	-0.5 (-0.9, 0)	-3.8 (-5, -2.9)	-2.9 (-3.9, -2.2)
			-2	0.8 (0.2, 1.6)	3.6 (2.5, 5)	2.7 (1.8, 3.8)	-1 (-1.4, -0.5)	-2 (-2.8, -1.5)	-3.1 (-4, -2.3)
	4	-1	-3	0.9 (0.3, 1.7)	3.7 (2.6, 5.1)	2.8 (1.9, 3.9)	-1 (-1.5, -0.5)	-3 (-4, -2.3)	-3 (-4, -2.2)
			-4	0.9 (0.3, 1.6)	3.8 (2.7, 5.1)	2.8 (1.9, 3.9)	-0.9 (-1.5, -0.4)	-3.8 (-5, -2.9)	-2.8 (-3.8, -2.1)
			-2	0.7 (0, 1.6)	3.4 (2.3, 5)	2.7 (1.7, 4)	-1.9 (-2.5, -1.4)	-2 (-2.8, -1.5)	-3 (-4, -2.2)
		-2	-3	0.9 (0.2, 1.7)	3.5 (2.4, 5)	2.7 (1.7, 3.9)	-1.9 (-2.6, -1.4)	-2.9 (-3.8, -2.1)	-2.9 (-3.9, -2.2)
			-4	0.9 (0.2, 1.7)	3.7 (2.5, 5.2)	2.7 (1.8, 3.9)	-1.9 (-2.5, -1.3)	-3.8 (-5, -2.8)	-2.8 (-3.8, -2.1)
			-2	1.9 (1.2, 2.9)	1.9 (1.2, 2.8)	2.9 (2, 4.1)	-0.5 (-0.9, -0.1)	-2.1 (-2.7, -1.5)	-3.1 (-3.9, -2.4)
		-0.5	-3	1.9 (1.2, 2.9)	1.9 (1.3, 2.8)	2.8 (2, 3.9)	-0.5 (-0.9, -0.1)	-3 (-3.8, -2.2)	-3 (-3.9, -2.3)
2	2		-4	1.9 (1.3, 2.8)	2 (1.3, 2.8)	3 (2.1, 4.1)	-0.5 (-0.9, 0)	-3.9 (-5, -3)	-2.9 (-3.8, -2.2)
			-2	1.9 (1.1, 2.9)	1.9 (1.2, 2.8)	2.8 (1.9, 4.1)	-1 (-1.4, -0.6)	-2.1 (-2.7, -1.5)	-3.1 (-4, -2.4)
		-1	-3	1.9 (1.2, 2.9)	2 (1.2, 2.8)	3 (2, 4.1)	-1 (-1.4, -0.5)	-3 (-3.9, -2.3)	-3 (-3.8, -2.3)
			-4	1.9 (1.2, 2.9)	1.9 (1.3, 2.8)	2.9 (2.1, 4.1)	-0.9 (-1.4, -0.5)	-3.8 (-4.9, -2.9)	-2.9 (-3.8, -2.2)

	-2	-2	1.8 (1, 3)	1.9 (1.1, 2.9)	2.8 (1.8, 4.2)	-1.9 (-2.5, -1.4)	-2 (-2.7, -1.5)	-3 (-3.9, -2.3)
		-3	1.9 (1.1, 3)	1.9 (1.1, 2.9)	2.9 (1.9, 4.2)	-1.9 (-2.5, -1.4)	-3 (-4, -2.3)	-3 (-3.9, -2.3)
		-4	1.9 (1.1, 2.9)	1.9 (1.2, 2.9)	2.9 (2, 4.2)	-1.9 (-2.5, -1.4)	-3.8 (-5, -2.9)	-2.9 (-3.8, -2.2)
3	-0.5	-2	1.8 (1, 2.7)	2.8 (1.9, 3.9)	2.8 (1.9, 3.9)	-0.5 (-0.9, 0)	-2.1 (-2.7, -1.5)	-3.1 (-4, -2.4)
		-3	1.9 (1.2, 2.9)	2.9 (2, 4)	2.8 (2, 3.9)	-0.5 (-0.9, -0.1)	-3 (-3.9, -2.3)	-3 (-3.9, -2.3)
		-4	2 (1.3, 2.9)	2.9 (2, 4)	2.9 (2, 4)	-0.5 (-0.9, 0)	-3.9 (-5, -3)	-2.9 (-3.8, -2.2)
	-1	-2	1.9 (1.1, 2.9)	2.8 (1.9, 3.9)	2.7 (1.8, 3.9)	-1 (-1.4, -0.5)	-2 (-2.7, -1.5)	-3 (-3.9, -2.3)
		-3	1.8 (1.1, 2.8)	2.9 (2, 4)	2.9 (2, 4)	-0.9 (-1.4, -0.5)	-3 (-3.9, -2.3)	-3 (-3.9, -2.3)
		-4	1.9 (1.1, 2.8)	2.9 (2, 4)	2.9 (2, 4)	-0.9 (-1.4, -0.5)	-3.8 (-4.8, -2.9)	-2.8 (-3.7, -2.1)
	-2	-2	1.8 (0.9, 3)	2.7 (1.7, 4)	2.7 (1.7, 4)	-2 (-2.5, -1.5)	-2 (-2.7, -1.5)	-3 (-3.9, -2.3)
		-3	1.8 (1, 2.9)	2.8 (1.8, 4.1)	2.7 (1.8, 4)	-1.9 (-2.5, -1.4)	-3 (-3.9, -2.3)	-3 (-3.9, -2.3)
		-4	1.8 (1, 2.8)	2.7 (1.8, 3.9)	2.7 (1.8, 3.9)	-1.9 (-2.5, -1.3)	-3.9 (-5, -2.9)	-2.9 (-3.8, -2.2)
4	-0.5	-2	1.8 (1, 2.7)	3.5 (2.5, 4.9)	2.6 (1.8, 3.7)	-0.5 (-0.9, -0.1)	-2 (-2.7, -1.5)	-3 (-3.9, -2.3)
		-3	1.7 (1, 2.6)	3.6 (2.6, 4.9)	2.6 (1.8, 3.7)	-0.5 (-0.9, 0)	-3 (-3.9, -2.3)	-3 (-3.8, -2.2)
		-4	1.8 (1.1, 2.6)	3.7 (2.7, 5)	2.8 (2, 3.9)	-0.4 (-0.9, 0)	-3.9 (-5, -3)	-2.9 (-3.8, -2.2)
	-1	-2	1.7 (0.9, 2.7)	3.5 (2.4, 4.9)	2.6 (1.7, 3.7)	-1 (-1.4, -0.6)	-2.1 (-2.8, -1.5)	-3.1 (-4, -2.4)
		-3	1.7 (1, 2.7)	3.6 (2.5, 5)	2.7 (1.8, 3.8)	-0.9 (-1.4, -0.5)	-2.9 (-3.8, -2.2)	-2.9 (-3.8, -2.2)
		-4	1.8 (1, 2.7)	3.6 (2.6, 5)	2.8 (1.9, 3.8)	-0.9 (-1.4, -0.5)	-3.9 (-5, -3)	-2.9 (-3.8, -2.2)
	-2	-2	1.6 (0.8, 2.8)	3.4 (2.2, 5)	2.6 (1.6, 3.8)	-1.9 (-2.5, -1.4)	-2 (-2.7, -1.5)	-3 (-3.9, -2.3)
		-3	1.7 (0.8, 2.7)	3.5 (2.3, 4.9)	2.6 (1.7, 3.9)	-1.9 (-2.5, -1.4)	-3 (-3.9, -2.3)	-3 (-3.9, -2.3)
		-4	1.7 (0.9, 2.7)	3.5 (2.4, 4.9)	2.6 (1.7, 3.8)	-1.9 (-2.5, -1.3)	-3.9 (-5, -2.9)	-2.9 (-3.8, -2.1)

2. Appendix A

Although the most common situation in practice is the evaluation of the association of potential covariates with the risk of being exposed to or suffering an event of interest, a second simulation was conducted to evaluate the performance of the proposed methodology when there are no covariates involved. In this case, different values of occurrences of the event of interest were considered (*m* = 50, 150, 250, 350, 450 for *n* = 500 and *m* = 550, 650, 750, 850 for *n*=1500). For each situation, the marginal posterior distributions of the parameters (estimated on the basis of Eq. (6)) have been summarized by their median and percentiles 2.5% and 97.5%.

Table S2 shows, for each combination of parameters, the average estimates and upper and lower limits of the 95% credibility intervals. It can be seen that the methodology can properly estimate the proportion of structural zeroes ($1-\omega$) in any situation but it is not possible to obtain proper estimates and narrow intervals for the proportion of sample zeroes ($1-p$) when the number of occurrences of the event of interest is low, because of the shape of the posterior distribution of p. This is the cost of using the non-informative uniform priors specified in Eq. (5).

Table S2. Simulation study results with no covariates.

n	m	$\hat{\omega}$ (95% CrI)	$\hat{p}$ (95% CrI)
	50	0.14 (0.09, 0.22)	0.71 (0.51, 0.98)
	150	0.39 (0.29, 0.49)	0.77 (0.58, 0.99)
500	250	0.49 (0.46, 0.50)	0.98 (0.91, 1.00)
	350	0.50 (0.49, 0.50)	1.00 (0.98, 1.00)
	450	0.50 (0.50, 0.50)	1.00 (0.99, 1.00)
	50	0.05 (0.03, 0.07)	0.71 (0.51, 0.98)
	150	0.14 (0.10, 0.21)	0.71 (0.51, 0.98)
	250	0.24 (0.16, 0.34)	0.71 (0.51, 0.98)
	350	0.33 (0.23, 0.47)	0.71 (0.51, 0.98)
1500	450	0.39 (0.30, 0.49)	0.77 (0.60, 0.99)
	550	0.43 (0.36, 0.50)	0.86 (0.72, 0.99)
	650	0.47 (0.42, 0.50)	0.93 (0.85, 1.00)
	750	0.49 (0.47, 0.50)	0.99 (0.95, 1.00)
	850	0.50 (0.49, 0.50)	1.00 (0.98, 1.00)

3. R code to reproduce analyses in section *Real data*

```
library(foreign)

library(rstan)

library(readr)

library(doParallel)

library(bayesZIB)
```

```
library(pscl)
library(robustbase)
library(MASS)
rstan_options(auto_write = TRUE)
options(mc.cores = parallel::detectCores())

nCores   <- detectCores()
registerDoParallel(nCores)

### Read the data
dades5 <- read.csv("Data/RealData_BayesZIB.csv")

### Plot prior and posteriors (Figure 2)
y <- dades5$sp_recod
### posterior distribution for w
post.w.noConst <- function(w)
{
  return((pbeta(w, sum(y)+1, length(y)-sum(y)+1)-pbeta(w/2, sum(y)+1, length(y)-
sum(y)+1))/w)
}

post.w <- function(w)
{
  Cp <- integrate(post.w.noConst, 0, 0.5)$value
  return(post.w.noConst(w)/Cp)
}

### posterior distribution for p
post.p.noConst <- function(p)
{
  return((pbeta(p/2, sum(y)+1, length(y)-sum(y)+1))/p)
}

post.p <- function(p)
{
  Cp <- integrate(post.p.noConst, 0.5, 1)$value
```

```
  return(post.p.noConst(p)/Cp)
}

posterior.w.cdf <- function(x)
{
  return(integrate(post.w, 0, x)$value)
}

posterior.p.cdf <- function(x)
{
  return(integrate(post.p, 0.5, x)$value)
}

### Estimates and credible intervals
uniroot(f=function(x){posterior.w.cdf(x)-0.025}, interval=c(0.001, 0.5))$root
uniroot(f=function(x){posterior.w.cdf(x)-0.500}, interval=c(0.001, 0.5))$root
uniroot(f=function(x){posterior.w.cdf(x)-0.975}, interval=c(0.001, 0.5))$root

uniroot(f=function(x){posterior.p.cdf(x)-0.025}, interval=c(0.5, 1))$root
uniroot(f=function(x){posterior.p.cdf(x)-0.500}, interval=c(0.5, 1))$root
uniroot(f=function(x){posterior.p.cdf(x)-0.975}, interval=c(0.5, 1))$root

### Figure 2
par(mfrow=c(2, 2))
### w prior density
x <- seq(0, 0.5, 0.00005)
prior_w <- dunif(x, 0, 0.5)
plot(x, prior_w, type="l", xlab=expression(omega), ylab="Prior density")

### w posterior density
posterior_w <- post.w(x)
plot(x, posterior_w, type="l", xlab=expression(omega), ylab="Posterior density")

### p prior density
x <- seq(0.5, 1, 0.00005)
prior_p <- dunif(x, 0.5, 1)
```

```
plot(x, prior_p, type="l", xlab="p", ylab="Prior density")

### p posterior density
posterior_p <- post.p(x)
plot(x, posterior_p, type="l", xlab="p", ylab="Posterior density")
dev.off()

### Model with covariates
set.seed(1234)
fit <- bayesZIB(sp_recod~gh|remplz, data=dades5, chains = 5,
                iter=5000, adapt_delta=0.999, max_treedepth=25)

print(fit$fit, pars=c("theta", "beta"))
ll <- sum(colMedians(as.matrix(fit$fit, pars="log_lik"))) ### Log-likelihood

### Comparison to ZIP / ZINB / OLS / Poisson / NB
summary(m1 <- zeroinfl(sp_recod~remplz|gh, data = dades5))
summary(m2 <- zeroinfl(sp_recod~remplz|gh, data = dades5, dist = "negbin"))
summary(m3 <- lm(sp_recod~remplz+gh, data=dades5))
summary(m4 <- glm(sp_recod~remplz+gh, data=dades5, family="poisson"))
summary(m5 <- glm.nb(sp_recod~remplz+gh, data=dades5))

### Model without covariates
set.seed(1234)
fit <- bayesZIB(sp_recod~1|1, priors=list(c(0,0.5), c(0.5,1)), data=dades5)

png("diagnostics.png", width=900)
  pairs(fit$fit, pars=c("theta", "beta"))
dev.off()

### Logistic regression models
summary(mod1 <- glm(sp_recod~gh, data=dades5, family="binomial"))
confint(mod1)
summary(mod2 <- glm(sp_recod~remplz, data=dades5[dades5$exposure==1, ],
family="binomial"))
confint(mod2)
```

R code to reproduce analyses in section *Simulation study*

```
library(bayesZIB)

library(doParallel)

library(WriteXLS)

options(mc.cores = parallel::detectCores())


nCores   <- detectCores()

registerDoParallel(nCores)


nsim <- 100


for (k in 1:nsim)

{

  n <- c(500, 1500) # Sample sizes

  theta0 <- c(-0.5, -1, -2)

  theta1 <- c(-2, -3, -4)

  theta2 <- -3

  beta0  <- c(0.5, 1, 2)

  beta1  <- c(2, 3, 4)

  beta2  <- 3

  resultat <- data.frame(expand.grid(n=n, theta0=theta0, theta1=theta1, 
theta2=theta2,

                                     beta0=beta0, beta1=beta1, beta2=beta2))


  genEsts <- function(i)

  {

    print(paste0("Simulation step ", i, " out of ", dim(resultat)[1]))

    n_sim <- resultat$n[i]

    theta0_sim <- resultat$theta0[i]

    theta1_sim <- resultat$theta1[i]

    theta2_sim <- resultat$theta2[i]

    beta0_sim <- resultat$beta0[i]

    beta1_sim <- resultat$beta1[i]

    beta2_sim <- resultat$beta2[i]


    ### Structural zeros (exposure)
```

```
x1 <- rnorm(n_sim)
x2 <- rnorm(n_sim)
z1 <- theta0_sim + theta1_sim*x1 + theta2_sim*x2
pr <- 1/(1+exp(-z1))
exposure <- rbinom(n_sim, 1, pr)

### Sample zeros (observed phenomenon)
x3  <- rnorm(n_sim)
x4  <- rnorm(n_sim)
z2  <- beta0_sim + beta1_sim*x3 + beta2_sim*x4
pr2 <- 1/(1+exp(-z2))
p    <- rbinom(n_sim, 1, pr2)
pres <- ifelse(exposure==0, 0, p)
df <- data.frame(exposure=exposure, pres=pres)

df$x1 <- x1; df$x2 <- x2; df$x3 <- x3; df$x4 <- x4
fit <- bayesZIB(pres~x1+x2|x3+x4, data=df, chains = 5,
                adapt_delta=0.999, max_treedepth=15, verbose=FALSE)
post_matrix  <- as.matrix(fit$fit, pars=c("theta", "beta"))
theta0_p50   <- median(post_matrix[, 1])
theta0_p2.5  <- quantile(post_matrix[, 1], 0.025)
theta0_p97.5 <- quantile(post_matrix[, 1], 0.975)
theta1_p50   <- median(post_matrix[, 2])
theta1_p2.5  <- quantile(post_matrix[, 2], 0.025)
theta1_p97.5 <- quantile(post_matrix[, 2], 0.975)
theta2_p50   <- median(post_matrix[, 3])
theta2_p2.5  <- quantile(post_matrix[, 3], 0.025)
theta2_p97.5 <- quantile(post_matrix[, 3], 0.975)
beta0_p50    <- median(post_matrix[, 4])
beta0_p2.5   <- quantile(post_matrix[, 4], 0.025)
beta0_p97.5  <- quantile(post_matrix[, 4], 0.975)
beta1_p50    <- median(post_matrix[, 5])
beta1_p2.5   <- quantile(post_matrix[, 5], 0.025)
beta1_p97.5  <- quantile(post_matrix[, 5], 0.975)
beta2_p50    <- median(post_matrix[, 6])
beta2_p2.5   <- quantile(post_matrix[, 6], 0.025)
```

```
    beta2_p97.5  <- quantile(post_matrix[, 6], 0.975)

    return(c(resultat$n[i], resultat$theta0[i], resultat$theta1[i], 
resultat$theta2[i],

             resultat$beta0[i], resultat$beta1[i], resultat$beta2[i],

             theta0_p2.5, theta0_p50, theta0_p97.5, theta1_p2.5, theta1_p50, 
theta1_p97.5, theta2_p2.5, theta2_p50, theta2_p97.5,

             beta0_p2.5, beta0_p50, beta0_p97.5, beta1_p2.5, beta1_p50, 
beta1_p97.5, beta2_p2.5, beta2_p50, beta2_p97.5))

  }


  system.time(dat.fin <- foreach(j=1:dim(resultat)[1], .combine=rbind) %dopar% 
genEsts(j))

  colnames(dat.fin) <- c("n", "theta0", "theta1", "theta2", "beta0", "beta1", 
"beta2",

                         "theta0_p2.5", "theta0_p50", "theta0_p97.5",

                         "theta1_p2.5", "theta1_p50", "theta1_p97.5",

                         "theta2_p2.5", "theta2_p50", "theta2_p97.5",

                         "beta0_p2.5", "beta0_p50", "beta0_p97.5",

                         "beta1_p2.5", "beta1_p50", "beta1_p97.5",

                         "beta2_p2.5", "beta2_p50", "beta2_p97.5")


  ### Excel exportation

  file_name <- paste0("../Results/simZIB_covs", k, ".xls")

  WriteXLS(as.data.frame(dat.fin), file_name)

}
```
